# Adaptive habits: understanding executive function and its development

**DOI:** 10.1016/j.tics.2025.10.016

**Published:** 2025-12-17

**Authors:** Jesse C. Niebaum, Allison Zengilowski, Benjamin Katz, Priti Shah, Yuko Munakata

**Affiliations:** 1 Center for Mind and Brain, Department of Psychology, University of California, Davis, CA, USA; 2 Department of Education and Human Services, Lehigh University, Bethlehem, PA, USA; 3 Department of Human Development and Family Science, Virginia Tech, Blacksburg, VA, USA; 4 Department of Psychology, University of Michigan, Ann Arbor, MI, USA

## Abstract

Executive functions (EFs) develop dramatically across childhood and predict important outcomes, including academic achievement. These links are often attributed to individual differences in EF capacities. However, individual difference accounts underemphasize contextual influences on EF. We propose a complementary perspective, the adaptive habits framework, which emphasizes how contextual factors support or hinder EF engagement in children. Contexts that support repeated EF engagement establish habits for engaging EF in similar contexts and in similar ways. Such habits, in turn, reduce the effort associated with engaging EF and thus increase the likelihood of deciding to engage EF in the future. We interpret empirical findings through the lens of adaptive habits, discuss the implications of this framework, and propose novel research approaches and interventions to support EF in children.

## Emphasizing contexts over capacities in EF development

Consider two children: one excels on assessments of academic achievement (see Glossary) and performs well on laboratory measures of cognitive flexibility, inhibitory control, and working memory. The other performs poorly on these same assessments. Why? Traditional theoretical frameworks of EF would attribute these variations in academic achievement to differences in EF capacities [1]. Performance on EF tasks that assess cognitive skills such as flexibility, inhibitory control, and working memory correlates with measures of academic success cross- sectionally and longitudinally [2–6]. These links are often interpreted causally – the EF capacities of children drive their learning and self-regulatory behavior in the classroom and beyond. In this view, environmental factors such as socioeconomic security and adversity, as well as individual factors such as genetic variations, influence the development of EF by shaping individual capacities [7,8].

These traditional frameworks underemphasize children’s remarkable sensitivity to contextual factors that shape their willingness to engage EF (Box 1) in the moment and across contexts. Accumulating evidence demonstrates that, although children may have the capacity to engage EF, they may adaptively not do so in some contexts because of their learning histories, sociocultural influences, and environment [9,10]. For example, the first child in our example might attend supervised preschool and play tablet-based games at home and thus gain practice with following adult instructions, solving abstract puzzles, and receiving rewarding feedback in these contexts. Such experiences may facilitate their decision to engage EF on abstract task-based EF assessments that typically involve arbitrary instructions from adults by making EF engagement habitual and less effortful in these contexts. By contrast, the second child may spend their time independently managing household chores, which involves EF skills such as shifting between tasks, holding multiple goals in mind, and planning but in more self-directed and practical contexts. These capacities may not be measured by abstract EF or academic assessments that differ from children’s everyday experience engaging EF and that have less contextual relevance for their everyday life [11–15].

Traditional frameworks have guided interventions aimed at increasing the EF capacities of children based on the reasoning that increased EF capacities will result in downstream benefits for academic achievement and associated life outcomes [16]. Such interventions often involve extensive practice on tasks that are used to assess EF or activities that require EF, such as music lessons, martial arts, or mindfulness training. However, meta-analyses have shown that the benefits of such interventions do not extend beyond improving performance on tasks that are identical or very similar to those used for training [17–22]. Despite extensive research efforts, diverted classroom time, and commercial enterprise, such interventions have shown limited success in achieving meaningful and long- lasting improvements in outcomes such as academic achievement [23]. Such findings underscore the need to rethink how EF interventions are conceptualized and implemented.

We propose a new framework, the adaptive habits framework, that emphasizes how learning histories, sociocultural influences, and environmental factors shape children’s decisions and willingness to engage, or not engage, EF. This framework offers an alternative and complementary understanding of EF, its development, and its role in academic achievement (see Box 2 for overlaps with and distinctions from other frameworks). Instead of targeting EF capacities in isolation, interventions that create contexts fostering children’s willingness to engage EF in ways that align with their academic and broader life goals should be more effective in improving EF and influencing real-world outcomes.

## The adaptive habits framework

**Figure 1.**
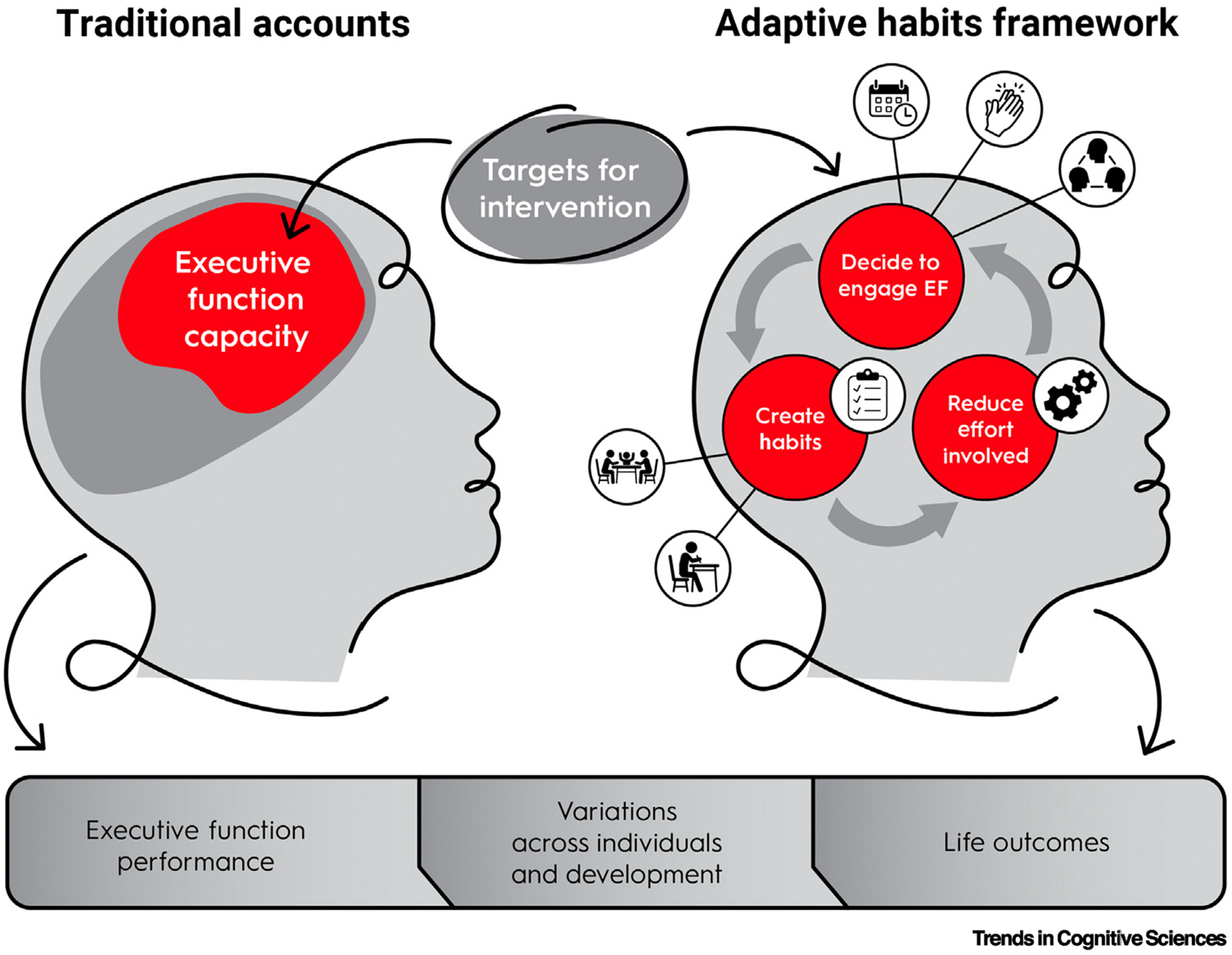
The adaptive habits framework. Traditional accounts of executive function (EF) focus on the capacity to engage EF (e.g., capacity limits on working memory span, inhibitory control, or cognitive flexibility). EF capacity is viewed as explaining performance on measures of EF, including variations in performance across individuals and groups, across development, and in life outcomes associated with those variations. EF capacity is also viewed as a primary target for intervention to support associated life outcomes. By contrast, the adaptive habits framework emphasizes that individuals can have the capacity to engage EF but decide whether or not to engage EF based on a variety of contextual factors (indicated by the icons linked to ‘decide to engage EF’, described next in counterclockwise order), such as what others around them are doing, actions that have been praised or rewarded in the past, and the predictability of their environment. These decisions to engage (or not engage) EF can support the development of habits around EF (indicated by the icons linked to ‘create habits’, described next in counterclockwise order), such as delaying gratification at mealtimes with others, persevering on challenging school assignments, and engaging control proactively in anticipation of needing it. These habits then make it easier to engage EF, which can in turn influence subsequent decisions to engage EF. In the adaptive habits framework, targets for intervention expand to include factors that influence decisions to engage EF, habits associated with EF, and the effort involved in engaging EF; these processes are viewed as explaining EF performance, variations across people, and associated life outcomes.

The adaptive habits framework (Figure 1) conceptualizes EF and its development as a reinforcing loop that operates across three components.

### (i) Deciding to engage EF

**Figure 2.**
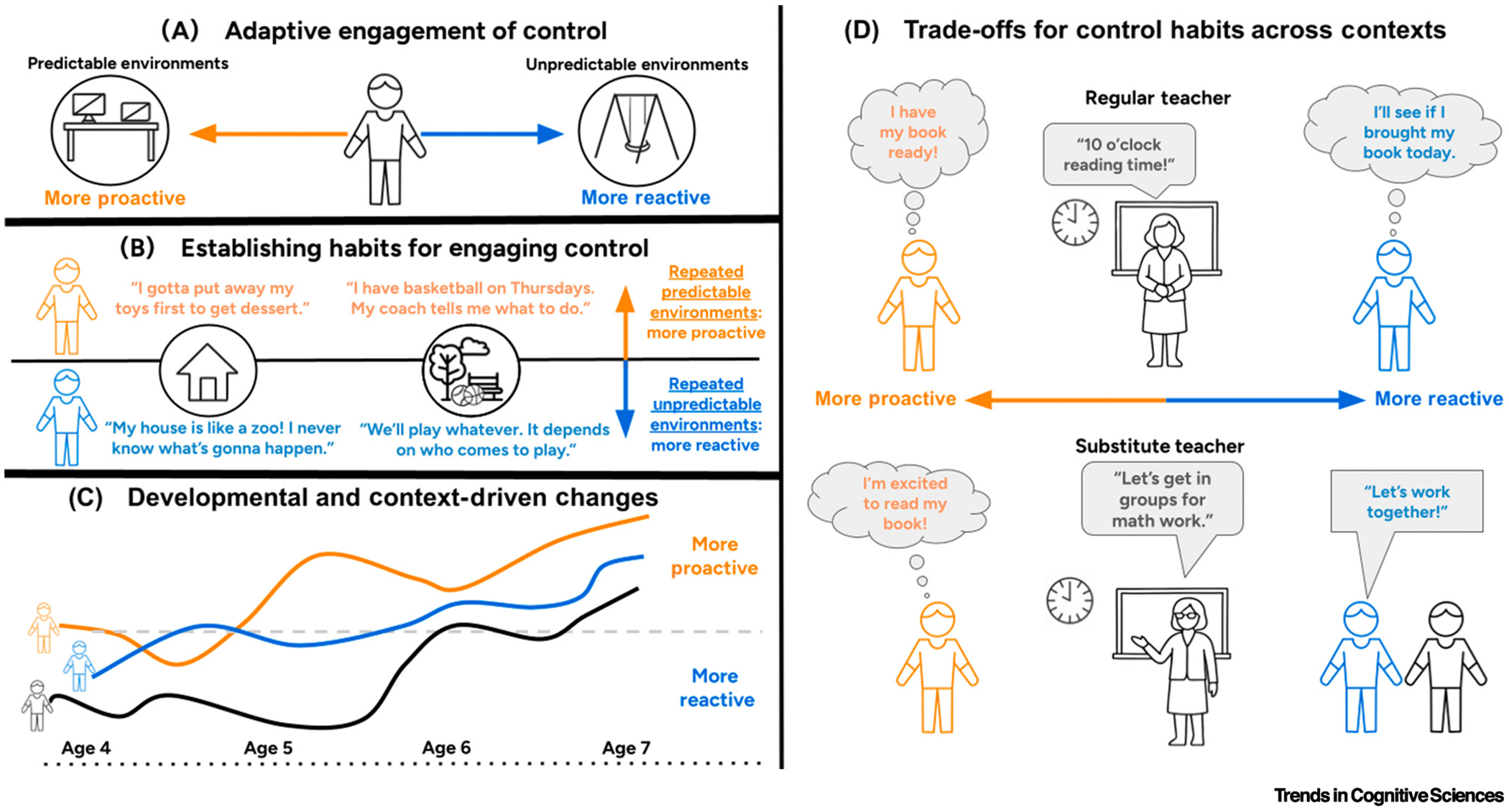
Contextually-driven decisions, habits, and trade-offs for engaging executive function (EF). (A) Individual children may shift between primarily engaging reactive or proactive control based on context. Contexts with higher predictability, such as classrooms with clear structure and planning, can encourage proactive engagement. Contexts with lower predictability, such as a busy playground at recess, can encourage reactive engagement. (B) Some children regularly and repeatedly experience more predictable contexts; such children should adapt to develop stronger habits for engaging proactive control. Other children regularly and repeatedly experience more unpredictable contexts; such children should adapt to develop stronger habits for engaging reactive control. (C) Children show increased engagement of proactive control with development, and variations in their trajectories reflect variations in their environmental contexts and neurocognitive development. (D) Habits for engaging proactive and reactive control have adaptive advantages or disadvantages, depending on contextual demands. Proactive control can be more efficient and effective in predictable contexts but less efficient and effective in unpredictable contexts. For example, proactive control may lead children to miss unexpected cues in the environment that signal a need to change behaviors, whereas reactive control can support adapting to unexpected cues in the environment.

Contextual factors influence the willingness of a child to engage EF. Such factors include extrinsic and intrinsic motivation, social and cultural norms, perceived effort relative to anticipated rewards, overlap between the current task and prior experiences, individual learning histories, and environmental factors [24,25]. For example, a child attending structured preschool that involves rulebased games may view gamified laboratory-based assessments as familiar and rewarding, whereas a child who is responsible for household chores may find that engaging EF is more worthwhile in more practical, autonomous contexts rather than on abstract tasks [11]. Differences in contextual support across environments, including at home and school, may also lead the same child to engage EF more willingly in one context over another (Figure 2). Children’s autonomy in deciding to engage EF could be inherently rewarding and increase the impact of outcomes following EF engagement [26]. Children need not be aware of their decisions surrounding EF engagement: increased willingness (or avoidance) could occur because of implicit motivations and automatic associations built up from prior experiences. Although not yet examined systematically, specific contextual factors that influence the willingness of children to engage EF likely vary throughout the course of development and across individuals and cultures [11,25].

### (ii) Creating habits

Repeated decisions to engage EF in consistent contexts (e.g., guided by specific cues, rewards, or goals) can create habits for engaging EF in similar contexts. Once habits have been established through repetition, engaging EF becomes increasingly less deliberate and more automatic [27,28]. For example, elementary school teachers often deploy cues (e.g., gestures, verbal cues) that signal to students a need to engage in self-regulatory behaviors such as listening or staying on task [29]. With repeated use, these cues can lead students to habitually regulate their classroom behavior.

### (iii) Reducing effort

Habits for engaging EF reduce perceptions of mental effort and support automaticity, thereby increasing willingness to engage EF [27]. For example, a child accustomed to completing homework in loud, unpredictable environments may find it easier to inhibit distractors on EF assessments than a child who is accustomed to completing homework in silent, predictable environments. A child accustomed to completing homework directly following school may find it easier to complete homework even when alternative activities are available.

Perceptions of mental effort guide decisions to engage EF. Adults and older children report that tasks high in EF demands are more aversive and effortful [30,31], select less- over more-demanding EF tasks when given the option [32,33], and expect higher incentives for completing more-demanding EF tasks [34–36]. When contextual factors and habit formation reduce the perceived effort associated with engaging EF, individuals will be more likely to decide to engage EF in the future.

The adaptive habits framework emphasizes prior learning and the sensitivity of children to current and past experiences, peers, and the broader environment. These contextual factors not only serve to support or hinder the willingness of a child to engage EF but also the habits and effort required to engage EF in the moment and in the longer term. In this view, environmental factors such as socioeconomic security and adversity also influence EF development in children by altering their decisions to engage EF, the habits they develop surrounding EF, and in turn, the mental effort associated with engaging EF. Different children, such as the two in our earlier example, may have similar EF capacities but differ in the extent to which they feel that engaging EF is ‘worth i’ based on the present context and its (mis)alignment with prior experiences.

Thus, assessments of EF capture not only individual differences in capacities but also the willingness to engage EF within a given task or context. This conceptualization of EF shifts focus from individual capacities to an interaction between context, habits, and effort. The adaptive habits framework also provides promising alternative targets for interventions: Instead of only targeting EF capacities, interventions should aim to create meaningful contexts and relevant incentives that encourage EF engagement [37].

We next review evidence supporting the components of the adaptive habits framework. We discuss how this framework provides explanations for differences in EF task performance across contexts and populations. Last, we interpret changes in EF and its association with academic achievement through the lens of adaptive habits. Predictions derived from this framework are discussed in Box 3.

## Deciding to engage EF

Instead of only reflecting fixed capacities, children’s engagement of EF is adaptive and contextdependent. Incentives, task demands, cultural and social norms, and daily socioemotional experiences can all contribute to fluctuations in the willingness of children to engage EF. Many EF assessments and interventions are gamified and incentivized to enhance motivation and engagement [38], two factors that are generally associated with improved performance [39]. For example, external rewards improve children’s accuracy and increase proactive control on sustained attention tasks [40]. Rewards improve performance on assessments of inhibitory control [41,42] and working memory [43]. Children also modulate their EF engagement based on reward likelihood, showing improved performance when the task context signals increasing probabilities of receiving a reward [44,45]. Other forms of reward, such as effort-based praise from adults, also increase EF engagement [46]. Parent praise for effort at age 2 predicts improvements in the inhibitory control of toddlers from age 2 to 4 years when controlling for inhibitory control performance at age2 and verbal ability at age 4 [47], which suggests that praise increases the willingness of children to engage EF.

Task demands also influence EF engagement. Children transition from primarily engaging reactive to proactive control at ~5–6 years of age [48–50], a shift that is typically attributed to improvements in working memory, which supports proactive goal maintenance [51,52]. However, 5-year-olds engage proactive control when engaging reactive control is made more difficult (e.g., by removing the sorting rules before children see the stimuli they need to sort) [53]. Instructing 4–6-year-olds to proactively prepare responses improves their performance and increases proactive control to levels comparable to those of 7–9-year-olds [54]. Children are also sensitive to task reliability: When cues about upcoming sorting rules are uninformative or unreliable, 6- and 9-year-olds tend to engage control reactively [55]. By contrast, when cues are reliable, these children tend to engage control proactively. Younger children are clearly capable of engaging proactive control but adaptively decide whether to do so based on context and task demands.

Similarly, children who appear to lack the capacity to delay gratification do delay when social incentives and expectations encourage delaying. For example, children wait longer when earning rewards for themselves than for a peer [56] and when the rewards of another child also depend on their waiting [57]. Social expectations also influence delaying: children wait longer when told that their teacher or peers will learn how long they waited [58] or that their in-group waited but an out-group did not [59,60]. Cameroonian children wait longer than German peers for a second treat; notably, wait time correlates with parent ratings of the importance of children developing respect for elders and other social factors [61]. Children are less likely to wait if they observe an experimenter lying to another person or failing to deliver promised art supplies [62–64]. Thus, children can adaptively decide to delay gratification (or not) based on whether they perceive that delaying is worthwhile or in line with culturally appropriate behavior.

The willingness of children to engage EF also depends on whether EF assessments reflect the cultural contexts in which children typically engage EF. For example, Yucatec Mayan children refuse to perform the day/night Stroop task to avoid making untrue statements and leave the room during delay of gratification tasks to avoid doing nothing [11]. Cultural differences in prioritizing speed/accuracy trade-offs can lead to different strategies when deciding how to engage control on speeded assessments of EF [65,66]. These findings highlight the need for culturally adaptive measures and interpretations of EF engagement [9,67]. What could appear to be low EF may instead reflect decisions not to engage EF when the context conflicts with cultural values, goals, and everyday experiences.

The willingness of children to engage EF also fluctuates with daily mood and the environment. Across a 7-day sampling period of 4–6-year-olds, most variance in parent-reported EF is within-children [68], which likely reflects environmental influences on EF engagement. The EF performance of adolescents fluctuates daily with self-reported mood and school belonging, with ~50% of the total variance in performance attributable to within-person variability [69]. These findings emphasize that EF task performance at least partially reflects willingness to engage EF.

The reviewed findings provide many targets for interventions to increase the willingness of children to engage EF. Interventions that provide meaningful and relevant incentives, create predictable and reliable task demands, and foster supportive social experiences have all shown promise in increasing the willingness of children to engage EF [70]. Cultural differences in what children perceive as motivating, relevant, or rewarding should factor into designs for EF interventions aimed at increasing their willingness [65].

## Establishing habits for engaging EF

Contextual factors that influence the willingness of children to engage EF in the moment may be stable across development, leading to repeated EF engagement, which establishes habits for engaging EF. For example, cross-cultural studies indicate that repeatedly delaying gratification can establish habits for waiting in similar contexts. In Japan, waiting until everyone has been served before eating is culturally normative [71]. Japanese children wait longer in delay of gratification tasks for food rewards than for gift rewards, which reflects culturally reinforced habits of waiting specifically around food [72]. US children show the opposite pattern: They wait longer for gift rewards than for food rewards, consistent with US cultural norms surrounding delayed giftopening. Japanese parents also report that their children have more experience waiting to eat and receive more encouragement to wait before eating compared with US parents, and these reported habits predict how long children wait for food but not gift rewards. Thus, cultural expectations and social reinforcement can shape the development of context-specific habits for delaying gratification.

Habits for engaging EF can also form within laboratory contexts. Five-year-olds, who typically engage control reactively, switch to engaging proactive control on a cued task-switching task when reactive control is less effective and continue to engage proactive control even after the task changes to allow more reactive engagement [73]. Similarly, adults and 10-year-olds, who typically engage control proactively, temporarily persist in engaging reactive control after completing tasks designed to discourage proactive control, even after the task context changes to allow proactive engagement [74].

Adults also develop habits based on learned reward histories. In a Stroop task, adults follow more control-demanding rules (color-naming) if features of the stimuli (ink color) were previously associated with rewards, even without continued rewards [75–77]. Similarly, adults rewarded for choosing to complete more-demanding EF tasks continue to select these tasks more often than adults who are rewarded for good performance, even when such decisions are no longer rewarded [78,79]. Habits for selecting more effortful tasks also generalize to other cognitive tasks that were never associated with reward. Reinforcing engagement of EF can thus create habits for engaging EF that influence task strategies and decisions.

These findings demonstrate that learning histories can establish habits for engaging EF, which can influence delaying gratification, the temporal dynamics of control, and task preferences. The history of an individual surrounding EF engagement can drive the formation of habits through multiple learning mechanisms. Rewarding EF engagement supports behavioral repetition through instrumental learning [80]. Associative learning processes can strengthen links between particular contexts or stimuli and EF engagement and thus make EF engagement more habitual over time [81–83]. These findings also have implications for interventions targeting EF. Pairing specific contexts with EF engagement should facilitate habitual EF engagement in similar settings. Habits formed in one context (e.g., waiting for food) can generalize to similar tasks when contextual cues and stimuli substantially overlap. Although the boundary conditions of such habit generalization are unknown, these findings demonstrate that EF interventions should reinforce repeated engagement through instrumental learning and strengthen associations between contexts and EF engagement.

## Reducing the effort of engaging EF

Decisions to engage with or avoid tasks that require EF are partially driven by subjective perceptions of effort, indicating that EF habits and associated reductions of effort influence EF engagement. When given a choice between tasks that require proactive or reactive control, adults select the proactive task, which they report as preferred, less effortful, and easier [84]. By contrast, 5- year-olds select the reactive task, which they report as easier and preferred. Paradigms that quantify the subjective value of mental effort have demonstrated that children who are more likely to conserve cognitive effort are also more likely to engage in reactive control than proactive control and report lower enjoyment for cognitively demanding activities [34]. These findings demonstrate that perceptions of effort differ across development and influence decisions about whether and how to engage EF.

The idea that habits for engaging EF reduce subjective effort broadly mirrors beliefs about the relationship between habits and effort more generally [85]. Reduced effort for engaging EF could occur in two ways: context-driven habits for engaging EF could (i) reduce the effort required in deciding how to engage EF (e.g., whether proactively or reactively); and/or (ii) increase skill and efficiency for engaging EF on specific tasks and in specific contexts.

Habits for delaying gratification appear to reduce the effortful engagement of EF that is required to wait for delayed rewards. Japanese children’s waiting for food rewards was unrelated to parent- reported inhibitory control, whereas their waiting for gift rewards was associated with inhibitory control [72]. Similar but non-significant patterns were observed for US children, except that inhibitory control better predicted US children’s waiting for food rewards, not gift rewards, in line with culturally specific norms. These findings suggest that habits reduce the effort required to engage EF and thus makes delaying gratification less effortful in contexts with established habits [27]. Japanese children also display less effort while waiting for food rewards than gift rewards (K. Yanaoka et al., unpublished) and tend to report that waiting for food rewards takes less work than waiting for gift rewards; this effect is greater for children with stronger habits of waiting to eat (K. Yanaoka et al., unpublished).

Habits are also linked to lower subjective effort for engaging self-control in adults [86]. Adults with stronger habits for healthy behaviors (e.g., diet, exercise, and sleep) report that abstaining from unhealthy behaviors feels less effortful than for adults with weaker habits. Such habits are also relevant in academic contexts. High school and college students with stronger study habits report a higher ability to study under difficult circumstances, have higher grade point averages (GPAs), and persist longer in college. Study habits were calculated as a function of the frequency and stability of relevant behaviors – an established index of habits that is also used in the study of healthy behaviors, suggesting that similar mechanisms may be at play: frequently repeated behaviors in similar contexts reduce subjective effort to support self control. However, subjective effort was measured only in the study on healthy behavior. Direct evidence that study habits reduce the effort associated with engaging self-control to support academic success is still needed.

One might ask whether EF is engaged at all after decisions to engage EF create habits that reduce the effort required to support goal-directed behavior. However, EF is still required for habitual goal-directed behaviors that might not seem to tap EF [87]. Patients with frontal lobe damage, who experience difficulties with EF, struggle with routine tasks, such as making breakfast or bedtime routines [88], indicating that these behaviors typically feel effortless because they are well- practiced, not because they no longer require EF. Similarly, goal-directed behaviors such as resisting temptations and updating working memory should engage EF even when they are supported by habits [89]. Future studies could explore this issue by assessing the engagement of frontal networks during EF processes as they become habitual.

## Age-related shifts in adaptive habits

As children develop, components of the adaptive habits framework likely shift in their influence or operation. Decisions to engage EF in early childhood may rely more on immediate environmental cues, whereas older children may be increasingly guided by self-directed goals and prior experiences with the value and subjective effort associated with engaging EF [90–92]. The same contextual cues may influence children’s motivation differently across age. For example, praise for persistence on challenging tasks may motivate younger children but signal failure for older children, who might view such praise as a substitute for success [93]. As children’s cognitive capacities increase with development, engaging EF may feel less effortful; for example, improvements in working memory may decrease the effort required for engaging proactive control, consistent with the observed correlation between working memory improvements and biases to engage control proactively across development [51]. Further, experiences with success and rewards, including developmental differences in reward sensitivities [94], may influence the perceived value of engaging EF. However, additional work is needed to understand systematic age-related changes in EF engagement, including throughout adulthood.

## Aligning contexts and prior experiences of engaging EF

The adaptive habits framework helps to explain differences in EF task performance across populations and contexts. Many relevant findings have been motivated by the hidden talents framework (Box 2): the adaptive habits framework offers complementary explanations focused on how underlying mechanisms influence EF engagement.

Adults who report growing up in unpredictable environments perform significantly better on shifting tasks when primed with thoughts of economic instability compared with a neutral condition and perform better than adults primed with thoughts of economic instability who report growing up in predictable environments [95]. This pattern does not extend to performance on an inhibitory control task. Similar patterns have been observed for working memory: adults who report growing up in unpredictable environments exhibit better working memory updating when primed with economic uncertainty compared with adults who report growing up in predictable environments, but worse in a control condition [96]. This benefit does not extend to working memory maintenance or retrieval. The hidden talents framework interprets these patterns as adaptive specializations to adversity. From the perspective of adaptive habits, repeated exposure to unpredictability could provide repeated practice with engaging specific aspects of working memory, which increases the likelihood and ease of engaging those aspects of working memory in similar contexts.

Using assessment stimuli relevant to the everyday experiences of children may reveal contextspecific habits of EF engagement. Children exposed to higher levels of poverty and violence perform worse on working memory and shifting tasks with abstract stimuli; however, these performance gaps disappear for working memory (but not shifting) when the tasks use familiar stimuli, such as money, faces, or public transportation [97]. The adaptive habits framework interprets this as evidence that past experiences have driven greater engagement of EF specifically for relevant items but not for abstract stimuli and have established habits for engaging EF for these relevant stimuli.

## Adaptive habits at school

The adaptive habits framework provides a lens for interpreting links between EF and academic outcomes. Performance on EF assessments has well-established correlations with academic measures, especially with standardized tests of achievement [98,99]. These correlations may partially reflect overlap in assessment content or context (e.g., individualized measures, taken alone with an adult) [100,101], although correlations are also observed between individual EF assessments and other academic outcomes such as GPA, as well as between standardized test scores and GPA [102,103]. These correlations may reflect not only EF capacities [1] but also the extent to which student habits for engaging EF align with the assessment contexts for EF and academic outcomes. When prior experiences and assessment contexts overlap, children may more readily engage EF in ways that support performance on EF and academic assessments.

The context-specific nature of EF engagement may also help to explain why performance on EF assessments conducted collectively in classrooms correlates more strongly with improvements in academic achievement across elementary years than assessments completed alone [104]. Similarly, classroom assessments of visual working memory are more strongly associated with standardized reading and mathematics test scores than individualized assessments [105]. EF assessments administered in classrooms likely better reflect the extent to which children are willing (and able) to engage EF for learning in school contexts. Assessing EF within the contexts in which they are engaged and needed, such as classrooms, can provide a better understanding of the links between EF and outcomes such as academic achievement.

Peers also play a crucial role in shaping how, and how much, children engage EF at school. Peers are the largest source of off-task behavior in the classroom, which negatively correlates with teacher-reported EF and academic achievement [106,107]. Peers also influence the EF of children over the longer term in ways that are consistent with the adaptive habits framework. In US preschools, class-wide averages of EF task performance predict individual improvements in EF task performance, self-regulation, and academic achievement across the early elementary school years [108–110]. These effects persist even after controlling for covariates that are commonly associated with EF, including teacher and classroom characteristics and classroom income, suggesting that improvements in EF across the academic years are caused by EF in peers. Similar patterns are observed in semi-rural populations in Ghana and urban populations in Brazil, where peer averages of EF and self-regulation predict individual increases in EF task performance and self-regulatory behaviors across elementary school years [111,112].

These findings have typically been interpreted from a capacity-based perspective (e.g., fewer classroom distractions enable children to perform at maximum ability) but are also compatible with adaptive habits: students who perform well on assessments of EF in group settings also signal to their peers through their behaviors more generally that engaging EF in the classroom is valued and socially encouraged, leading children to repeatedly engage EF in the classroom. Over time, this repeated practice in engaging EF over the academic year drives improvements in EF and self-regulation not only by increasing children’s decisions to engage EF but also by developing habits for engaging EF within classroom contexts that improve efficiency in engaging EF and by decreasing the effort required to self-regulate within the classroom (Box 4 for cautions about attempts to instill EF in school contexts).

## Concluding remarks

Our aim in reviewing the literature that supports the adaptive habits framework is not to refute traditional capacity-based models of EF. Instead, we aim to highlight how capacity-based frameworks are insufficient to fully explain EF and its development. Extensive empirical evidence supports reliable developmental transitions in EF capacities and stable individual differences that predict a range of positive life outcomes. These developmental changes and individual differences may reflect both individual EF capacities and the acquisition of adaptive habits that shape how children engage EF as they develop. Similarly, within-individual variability in EF task performance may reflect both fluctuations in EF capacities, which can be influenced by sleep or hunger, and factors related to the willingness to engage EF. Although many questions remain (see Outstanding questions), the adaptive habits framework provides a mechanistic explanation for both between- and within-individual variability that moves beyond capacities alone.

Why do children (such as the two in our opening example) differ in their academic and EF task performance? The reviewed evidence demonstrates that such differences should be viewed as a product of distinct learning histories, sociocultural influences, and environmental contexts instead of solely as differences in EF capacities. The adaptive habits framework emphasizes how contextual factors influence children’s decisions to engage EF and how such engagement (or its absence) supports the development of habits that make it easier (or harder) to engage EF in similar contexts or for similar rewards in the future. Thus, two children may have the same EF capacities; however, one child may perform better on standard measures of EF because these measures better align with how the child has practiced engaging EF in the real world and how their behaviors have been rewarded and reinforced, which in turn reduces the mental effort needed for engaging EF. The adaptive habits framework thus identifies these contextual factors as promising targets for future research on EF as well as for interventions to support the EF and academic achievement of children.

## Highlights

Traditional views of executive function (EF) and its development focus on capacities and how environmental and individual factors influence EF capacities.

Recent evidence demonstrates that performance on assessments of EF reflects not only children’s EF capacities but also their willingness to engage EF during those assessments. This willingness to engage control varies according to individual learning histories, sociocultural influences, and environmental factors.

We leverage these empirical findings to propose a complementary framework for understanding EF and its development, as well as its links with outcomes such as academic achievement that emphasizes contextual influences on children’s decisions to engage EF and identifies new targets for interventions to support EF and associated outcomes.

## Box 1. What is the willingness to engage EF?

Willingness to engage EF refers to the propensity of an individual to allocate cognitive effort toward tasks that require EF, which can vary across contexts and individuals. Although separating willingness to engage EF from EF capacity is challenging, they are conceptually and psychometrically distinct, with indices that capture the subjective value of engaging EF only weakly correlating with EF task performance [34,36]. Similarly, paradigms that capture preferences for avoiding EF engagement show only small correlations with task performance [32,33]. Such correlations suggest that performance on standard EF measures captures both capacities and willingness and that willingness to engage EF varies meaningfully across individuals.

Willingness to engage EF draws on expected value of control theory, which proposes that individuals allocate cognitive control when the expected benefits outweigh perceived costs, incorporating the subjective costs of engaging control, expected rewards, and the likelihood of obtaining rewards when engaging control [113]. Willingness to engage EF is thus a motivational construct that is sensitive to rewards, task demands, and perceptions of effort. Willingness to engage EF also builds on learned value of control theory, which proposes that individuals learn the value of engaging control through prior experiences [77]. When engaging control leads to rewards, individuals develop associations between task features and rewards, which increases the likelihood of engaging control in similar future contexts. Context-specific associations between engaging control and reward can be shaped by metacognitive, sociocultural, and environmental factors that vary within and across individuals [68,93,114]. Together, these influences shape willingness to engage EF.

Importantly, the adaptive habits framework also proposes that habits for engaging EF, which have been established through repeated engagement, can override real-time cost–benefit analyses about the expected value of control, leading to automatic control engagement in familiar contexts, even when task demands are high or anticipated rewards are low [75,76]. In these contexts, willingness to engage EF is not the result of explicit decision-making; instead, habit formation can make willingness to engage EF a more automatic and less willful process.

## Box 2. Adaptive habits in relation to other frameworks

Several frameworks have shaped the development of the adaptive habits framework, in part through collaborative efforts. Similarities and distinctions between frameworks include:

Expected value of control (EVC)

EVC theory [113,115] posits that the dorsal anterior cingulate cortex (dACC) determines whether and how much control to engage, based on the expected benefits and costs of engaging control. The adaptive habits framework shares with EVC a focus on decisions to engage control, such that low levels of control need not imply low EF capacities but can instead reflect decisions driven by context. The adaptive habits framework extends beyond EVC by focusing on the developmental context and on how such decisions to engage EF can shape habits of EF and reduce associated effort. These processes influence the benefit–cost calculations for engaging control described by EVC.

Hidden talents framework

The hidden talents framework emphasizes how children experiencing adversity can develop stress-adapted skills that are often overlooked in deficit models of stress and development [116,117]. Adaptive habits shares with hidden talents and other frameworks [118] the view that the thoughts and actions of children reflect their learning and adaptation to their environments – these adaptations can manifest as strengths or deficits depending on the match between earlier environments and the current context and depending on who is labeling or judging the adaptations. Thus, the ‘adaptive’ aspect of adaptive habits reflects not only that habits lead to increased automaticity and reduced effort but also that habits are formed as adaptations to the environment. Adaptive habits extends beyond hidden talents in our focus on the underlying cognitive mechanisms that support the adaptations children show in their engagement of EF. Thus, although interventions derived from the hidden talents framework might focus on designing contexts that match the prior experiences of children to best engage and reveal EF capacities, interventions derived from the adaptive habits framework focus on increasing decisions to engage EF in goal-relevant contexts and on creating habits that make engaging EF easier in those contexts.

Skills-based frameworks

Skills-based frameworks posit that EF develops via ‘skills in using control in the service of specific goals’ rather than via domain-general components such as inhibition, shifting, and updating working memory [119] (see also [120,121]). Collaborative work with one of the skills-based proponents [59,60,122] expanded our understanding of social context in the EF of children (see also [61,123,124]). We concurrently wrote an integrative review that discusses the implications for conceptualizing, measuring, and supporting EF across development [25]. This shared history has contributed to overlap in focusing beyond EF as stable internal capacities. However, in contrast with strictly skill-based perspectives, the adaptive habits framework focuses on how context influences children’s engagement and subsequent development of domain-general EF, which can be understood in terms of the underlying mechanisms, computations, and cognitive processes that can be meaningfully delineated [125–127].

## Box 3. Predictions derived from the adaptive habits framework

The adaptive habits framework makes testable predictions about how experiences influence EF engagement and development, as well as about relationships between EF and educational and other life outcomes. First, repeated EF engagement should establish habits that make future EF engagement less effortful and more likely. We discuss supporting evidence in the context of proactive/reactive control and delay of gratification in the main text. Additional predictions include: children raised in unpredictable environments should be more likely to rely on reactive control because engaging control proactively in anticipation of future demands may rarely pay off, and resulting habits for reactive control should make engaging control reactively less effortful. Conversely, children raised in predictable environments should exhibit stronger biases for engaging control proactively and find doing so less effortful [128]. Similarly, early exposure to environmental unpredictability should reduce the willingness of children to delay gratification, especially in contexts where the reliability of delayed rewards is uncertain.

Second, performance on EF assessments should best predict life outcomes such as academic achievement when the assessment context closely matches the outcome context, provided that the assessment context still taxes EF [129]. For example, proactive control should better predict academic success in structured, predictable school environments. Conversely, reactive control should better predict success in more dynamic and less predictable class environments. EF assessments that involve numerical content should better predict achievement in mathematics than non-numerical and abstract tasks [100]. Delaying gratification should predict life outcomes in context-specific ways: delaying for monetary rewards should predict financial outcomes better than delaying for food or gifts.

Third, interventions to support the EF of children in school settings should be more effective when they reinforce children’s decisions to engage EF and provide meaningful, repeated opportunities to do so. Such repeated engagement should facilitate habit formation and reduce the effort associated with engaging EF over time. For example, games designed to boost the willingness of students to engage EF in fun contexts with peers, such as Spark Math games, should support the establishment of habits for engaging EF with mathematics content by motivating students to play repeatedly and reducing the effort involved with such engagement of EF [38,130]. Further work will be necessary to test these predictions more systematically, including the predicted impacts on student perceptions of effort.

## Box 4. What the adaptive habits framework does and does not imply for school contexts

The adaptive habits framework proposes that contextual modifications can serve as effective interventions to increase EF engagement in academic contexts. It does not imply that regimented, harsh, or strict environments are intrinsically beneficial for students, or that engaging EF effectively always means that the student is still, silent, and working independently.

Children more successfully engage EF in the classroom when they feel a sense of belonging at school [69,131]. School environments with high belongingness not only value student self-determination and autonomy but also help students to form positive, trusting relationships with teachers [132,133]. Student perceptions of teacher support and caring are the strongest predictors of school belongingness [134]. Interventions directed at improving the belonging-centered instruction practices of teachers predict student engagement and agency in mathematics [135]. By contrast, schools with punitive disciplinary practices in response to minor infractions such as disruptive behavior have a negative impact on students’ sense of belonging at school [136]. Feelings of school belonging are associated with motivation, including the perceived value of schooling, academic motivation, and effort [132,137]. Factors that support belongingness in school, such as social support, may help to explain individual differences in performance on measures of EF and why differences in performance on EF tasks predict positive life outcomes beyond a capacity-based understanding [61,124].

Because decisions to engage EF also depend on the effort required, classroom contexts that place extraneous demands on EF may reduce the willingness of students to engage EF in the service of learning (in addition to potentially reducing their capacity to engage EF). For example, a classroom that requires students to be still and silent for extended periods taxes inhibitory control over the natural tendencies of students to move and talk, such that students may opt to not put forth the additional effort associated with the inhibitory control that is necessary to solve mathematical problems. Other environmental factors can also place extraneous demands on student EF and reduce their likelihood of engaging EF for school work, such as temperatures that are too hot or too cold, noise, air pollution, or dilapidated buildings [138,139]. In addition to these school environment factors, students whose basic biological needs are not met – nutrition, sleep, physical activity, safety, exposure to nature, and play, or who are coping with anxiety, depression, or stress, or trauma – may find it more effortful to engage EF. These considerations highlight the need for supportive, meaningful, and motivating environments to foster EF engagement [140].

## Outstanding questions

How can standardized measures of EF be adapted to better capture the real- world skills of children and reflect real- world contexts such as the classroom environment? How can classroom environments be designed to better support the development of EF skills that are relevant to the real world?

How well do more naturalistic and relevant measures of EF predict different aspects of academic achievement, especially measures beyond standardized tests such as student engagement, graduation rates, and course selection? What are the implications for theorizing about the causal role of EF in academic achievement?

What contextual factors most influence the willingness of children to engage EF, and how does the influence of these factors change with age? How do current contextual factors interact with the prior experiences of children to influence EF engagement?

Incentives, peers, and environments may implicitly encourage EF engagement by children without their awareness. How do such factors generalize to the explicit decisions of children to engage EF, for example by taking on more challenging tasks or coursework?

What are the boundary conditions for transfer following contextually driven interventions designed to increase EF engagement in children? What factors predict whether such interventions generalize to other contexts? How can contextually driven interventions be designed to operate at scale?

To what extent does variability in EF performance within an individual, both across time and context, explain academic achievement?

How does the malleability of habits surrounding EF change across development? Are EF habits more easily established or more durable at particular ages?

How do developmental changes in sensitivity to cognitive effort or expected rewards influence decisions to engage EF?

How does the engagement of neural mechanisms that support EF change as EF processes become more habitual?

## Glossary

Academic achievement: indicators of academic success, frequently assessed by performance on standardized tests (e.g., in research on the links between EFs and academic achievement). Other measures may better capture academic achievement in context, such as grade point average (GPA), student involvement, retention, degree completion, and curriculum tracks. All measures can vary across contexts and have potential for racial, cultural, socioeconomic, and gender biases
Belonging-centered instruction: a pedagogical framework for creating inclusive learning environments through interpersonal interactions and instructional practices that promote the active inclusion, achievement, identification, and empowerment of students
EF capacities: the upper-limit ability of an individual for specific EF processes given optimal conditions
Executive functions (EFs): an umbrella term used to refer to cognitive processes that support flexible, goal- directed thought and behavior, including cognitive flexibility, inhibitory control, working memory, reasoning, and planning
Habit: a learned and repeated behavior in specific contexts (e.g., EF habits refer to learned and repeated behaviors for engaging EFs in specific contexts or for specific goals)
Interventions: programs or training that aim to improve EF in children and downstream outcomes such as academic achievement, often through repeated practice on EF assessments or activities that are believed to require EF. We also use intervention to include contextual and environmental changes that may increase EF engagement by children
Mental effort: the expenditure of cognitive resources towards a specific cognitive task, particularly tasks that require EF
Task-based assessments: measures that require children to complete typically arbitrary tasks that are often administered as tabletop games or digitally on tablets or computers. EFs are assessed via metrics of the performance of children, such as response times and accuracy
Willingness to engage EF: the propensity to engage mental effort in tasks that require EF. The willingness of an individual to engage EF and their EF capacity operate together to determine performance on EF assessments
